# Understanding Musical Predictions With an Embodied Interface for Musical Machine Learning

**DOI:** 10.3389/frai.2020.00006

**Published:** 2020-03-03

**Authors:** Charles Patrick Martin, Kyrre Glette, Tønnes Frostad Nygaard, Jim Torresen

**Affiliations:** ^1^Research School of Computer Science, Australian National University, Canberra, ACT, Australia; ^2^Department of Informatics, University of Oslo, Oslo, Norway; ^3^RITMO Centre for Interdisciplinary Studies in Rhythm, Time and Motion, University of Oslo, Oslo, Norway

**Keywords:** musical performance, interface, mixture density network (MDN), recurrent neural network (RNN), creativity, predictive interaction, embodied performance

## Abstract

Machine-learning models of music often exist outside the worlds of musical performance practice and abstracted from the physical gestures of musicians. In this work, we consider how a recurrent neural network (RNN) model of simple music gestures may be integrated into a physical instrument so that predictions are sonically and physically entwined with the performer's actions. We introduce EMPI, an embodied musical prediction interface that simplifies musical interaction and prediction to just one dimension of continuous input and output. The predictive model is a mixture density RNN trained to estimate the performer's next physical input action and the time at which this will occur. Predictions are represented sonically through synthesized audio, and physically with a motorized output indicator. We use EMPI to investigate how performers understand and exploit different predictive models to make music through a controlled study of performances with different models and levels of physical feedback. We show that while performers often favor a model trained on human-sourced data, they find different musical affordances in models trained on synthetic, and even random, data. Physical representation of predictions seemed to affect the length of performances. This work contributes new understandings of how musicians use generative ML models in real-time performance backed up by experimental evidence. We argue that a constrained musical interface can expose the affordances of embodied predictive interactions.

## 1. Introduction

**Figure 1:**
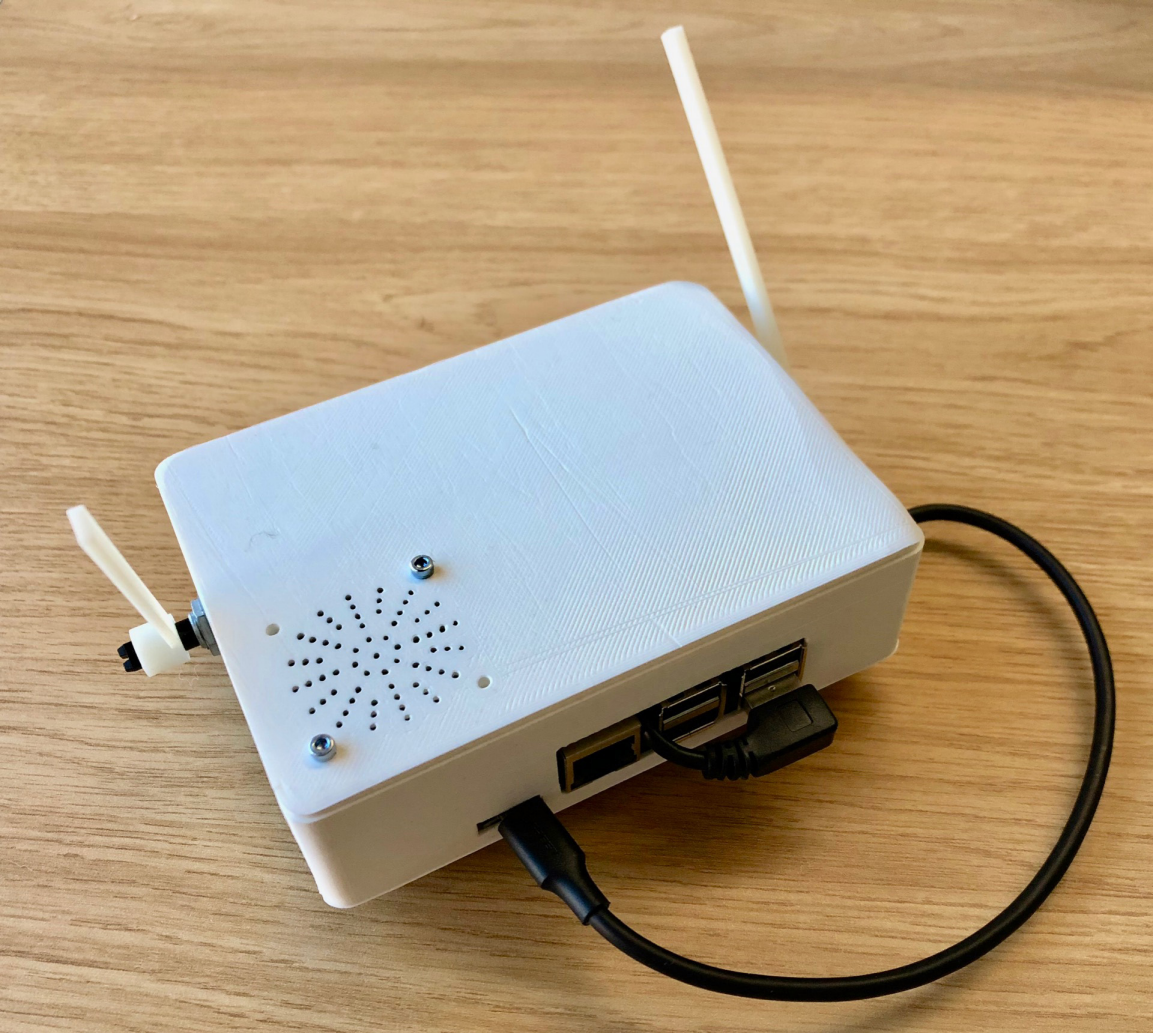
The Embodied Music Prediction Interface (EMPI) prototype. The system includes a lever for a performer's physical input (left side) and a motor-controlled lever for physical output, a speaker, and Raspberry Pi. This system represents a minimum set of inputs and outputs to experiment with embodied predictive interaction. A demonstration video can be viewed in the Supplementary Material.

It is well-known that music is more than just what you hear. Movements, or gestures, also contribute to musical communication (Jensenius et al., 2010). Most acoustic music performance involves control gestures to operate instruments, but performers also use expressive auxiliary gestures to communicate musical expression (Broughton and Stevens, 2008). In contrast, machine-learning models of music often exist outside the world of physical performance with music represented symbolically or as digital audio, both forms abstracted from musicians' physical gestures. If these models are to be applied in real-time musical performance, then it is crucial to know whether performers and listeners understand predicted musical information and how they use it. In this work, we consider how a recurrent neural network (RNN) model of simple music gestures may be integrated into a physical instrument so that predictions are sonically and physically entwined with the performer's actions. Our system, the embodied musical prediction interface (EMPI, see Figure 1), includes a lever for physical input from a performer, and a matching motorized lever to represent predicted output from the RNN model. We use this interface to investigate how performers can make use of musical machine-learning predictions in real-time performance, and whether physical representations might influence their understanding of such an instrument.

Rather than predicting symbolic music, such as MIDI notes, our RNN model predicts future musical control data—the physical positions of the EMPI's lever—in absolute time. These predictions can thus be represented both through the sound produced by predicted movements as well as through physical actuation of these control elements. The goal is to train a machine-learning model that can improvise on a musical instrument directly, rather than compose notes. To examine the potential of this idea, our EMPI system simplifies musical interaction to the barest requirements: just one dimension of continuous input and output which both control the pitch of a synthesized sound. By reducing the musical prediction problem, we seek to expose the performers' understanding of and adaptation to a musical ML system.

The EMPI system includes a single-board computer for machine-learning calculations and synthesis, one lever for physical input, one for actuated physical output, and a built-in speaker. It is completely self-contained, with power supplied by a USB power bank. The machine-learning model is a mixture density RNN trained to predict the performer's next physical input action and the time at which this will occur (Martin and Torresen, 2019). The system includes three different models: one trained on a corpus of human-sourced performance data; one trained on synthetically produced movements; and one trained on noise, or movements that are uncorrelated in time. Although multiple interaction designs could be possible, we focus here on applying predictions to continue a performer's interactions (Pachet, 2003), or to improvise in a call-and-response manner.

Embedded and self-contained instruments are important current topics in digital musical instrument design (Moro et al., 2016); however, these instruments usually do not include predictive capabilities. On the other hand, musical AI is often focused on composition using high-level symbolic representations (e.g., Sturm and Ben-Tal, 2017), and not the interactive or embodied factors (Leman et al., 2018) of music perception and creation. In this work, an embedded instrument design is combined with a novel, embodied approach to musical AI. This combination of embodied musical prediction with interaction allows us to explore musical AI within genuine performance environments, where movement is entangled with sound as part of musical expression.

We evaluated the success of this system through examination of generated data from these trained models as well as through a study of 72 performances made with this system under controlled conditions with 12 performers. This evaluation sought to identify whether the actions of the different predictive models are understandable to the performers, and whether they perceive useful musical relationships between their control gestures, and the model's response. We also investigated whether embodied interactions with this system's physical output improves or distracts from these understandings.

Our survey findings show that, of the three models, the performers assessed EMPI's human model as most related to their performance, most musically creative, more readily influenced and more influential on their playing than the other models. However, interviews with participants revealed they also saw value in the synthetic and even noise model based on their interactive affordances and musical styles. While performers were split on opinions regarding the physically embodied response lever, the length of improvisations suggests that the lever did effect their perceptions of the model's actions. Our study has demonstrated that a constrained, ML-enabled musical interface can afford a variety of creative performance styles. The performer's understanding of the different ML models seems to have a significant bearing on how they interact with the interface. We argue that physically actuated indicators, although potentially distracting for some performers, can expose the actions of an embodied music model, and encourage users to explore new ways of performing.

## 2. Background

Musical instruments are not typically predictive; instead, definitions of interactive music systems focus on behavior in reaction to gestural input (Rowe, 1993). The advent of electronic musical instruments including powerful computers has allowed experiments with instruments that are able to make intelligent use of the musical context in which they are used. This has been discussed since at least the early 1990s (Pressing, 1990), but has been extended in recent years with the development and popularity of accessible machine learning frameworks for understanding physical gestures in performance (Fiebrink, 2017). Artificial intelligence techniques can imbue a musical interface with a kind of self-awareness (Lewis et al., 2016; Nymoen et al., 2016), allowing them to act predictively, rather than in reaction to a performer.

**Figure 2:**
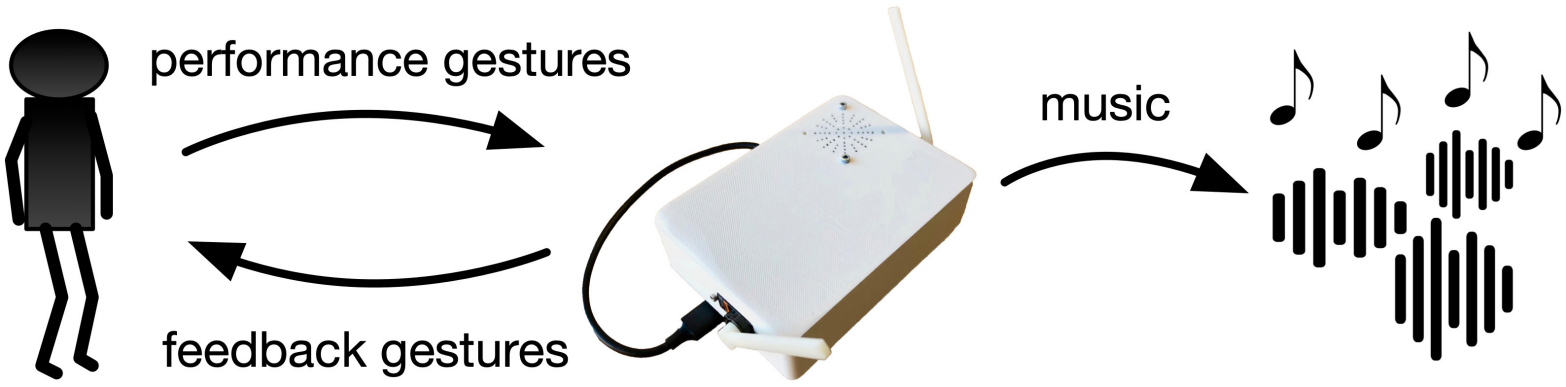
Typical musical instruments translate physical gestures into musical sounds. A predictive instrument can guess future gestures and use this knowledge to provide continuous sonic output and physical feedback to the performer.

The question of how to make best use of musical predictions, particularly from a performance perspective, remains open. Present work in musical deep neural networks is often focused on symbolic music generation (Briot et al., 2020), on the modification (Roberts et al., 2018) or in-filling (Huang et al., 2017) of given musical sequences, and creating musical digital audio (Engel et al., 2019). Examples of these neural networks have recently been embedded into digital audio workstation software to aid users during music composition (Roberts et al., 2019). Predictions are therefore used to make *more* music, or *better* music. We do not stray far from this characterization in the present work, but rather consider musical data to include gestural feedback, as well as more typical notes and sounds. Where a typical musical interface maps gestures into sounds, a predictive interface can also map current gestures into future gestures and represent these gestures themselves as well the sounds they might produce (see Figure 2).

Music has many representations, including lead sheets, scores, and recorded audio with varying levels of specificity over the musical work recorded (Davies, 2005). The machine learning models mentioned above have focused on generating music represented either symbolically (e.g., as MIDI notes), or as digital audio, a more-or-less finalized representation. In this work, we use control gestures to represent musical performance; a format that is more open than digital audio, but more specific than MIDI notes, especially in terms of precise expression. As argued in section 1, control and auxiliary gestures are important parts of musical performance (Jensenius et al., 2010). Further, an embodied view is required to understand how we perceive and perform music (Leman et al., 2018). Some machine learning models do predict embodied representations of artistic data. For instance, *SketchRNN* predicts pen movements to draw images (Ha and Eck, 2017), and *SPIRAL* generates instructions for a paint program to generate realistic images (Ganin et al., 2018). This concept has also been applied to musical sketches in *RoboJam* (Martin and Torresen, 2018), and the IMPS system (Martin and Torresen, 2019), which applied similar mixture density RNNs as in the present research to predict movements on a touchscreen or of arbitrary numbers of control values through time. One field where embodied music is crucial is musical robotics (Bretan and Weinberg, 2016), although physical motions in this field are usually not the direct predictions of an ML system, but programmed in response to decisions to actuate certain notes on an acoustic instrument.

The EMPI system in this work is an example of an embedded and self-contained computer music interface. Handheld and self-contained electronic instruments, such as Michel Waisvisz' *CrackleBox* (Waisvisz, 2004), the toy *Stylophone* (McNamee, 2009), or Korg's more recent *monotron* synthesizers have been popular since the late 1960s. While most computer music instruments involve a laptop computer externally connected to a controller interface, Berdahl and Ju (2011) argued that it was advantageous to embed a single-board computer (SBC), such as a Raspberry Pi inside the musical instrument to create an integrated and portable musical instrument. The resulting *Satellite CCRMA* system used a Raspberry Pi with a USB-connected microcontroller (Berdahl et al., 2013). The *Bela* system (Moro et al., 2016) developed this idea, with an integrated hardware extension to the Beaglebone Black platform providing an embedded instrument platform with high audio and sensor performance (McPherson et al., 2016).

Apart from technical advantages, embedded instrument designs can be artistically advantageous in terms of enabling exploration through physical manipulation (Reus, 2011) and even live hardware hacking (Zappi and McPherson, 2014). Self-containment can also enable new research methodologies. Gurevich et al. (2012) explored a constrained self-contained musical interface. In this case, the self-contained nature of the device allowed it to be distributed to participants and explored by them on their own terms.

So far, there are few examples of embedded computer music interfaces that include music prediction ANNs. This is despite significant interest in ML-prediction on internet of things (IoT) or edge computing platforms (Ananthanarayanan et al., 2017). In one of the only present examples, Næss and Martin (2019) demonstrated an LSTM-RNN-driven embedded music generator based on a Raspberry Pi. This work showed that RNN prediction is practical on an embedded system, and the resulting self-contained interface allows the music generation system to be examined by musicians. In the present research, we also use a Raspberry Pi as the embedded computing platform for an RNN-based musical prediction system. This work goes further by exploring musical predictions at the gestural, rather than symbolic level of representation. Our system embeds a predictive model in a system with physical, as well as sonic output. This allows us to examine both musical expression and predictive interaction in a real-time performance situation.

## 3. System Design

Our Embodied Musical Predictive Interface (EMPI), shown in Figure 1, is a self-contained musical interface. EMPI is a highly constrained musical interface, with only one dimension of continuous input. The EMPI's matching physical output allows it to represent the embodied predictive process to a human user. Its self-contained form-factor allows musicians to explore and integrate predictive musical interaction into different scenarios.

**Figure 3:**
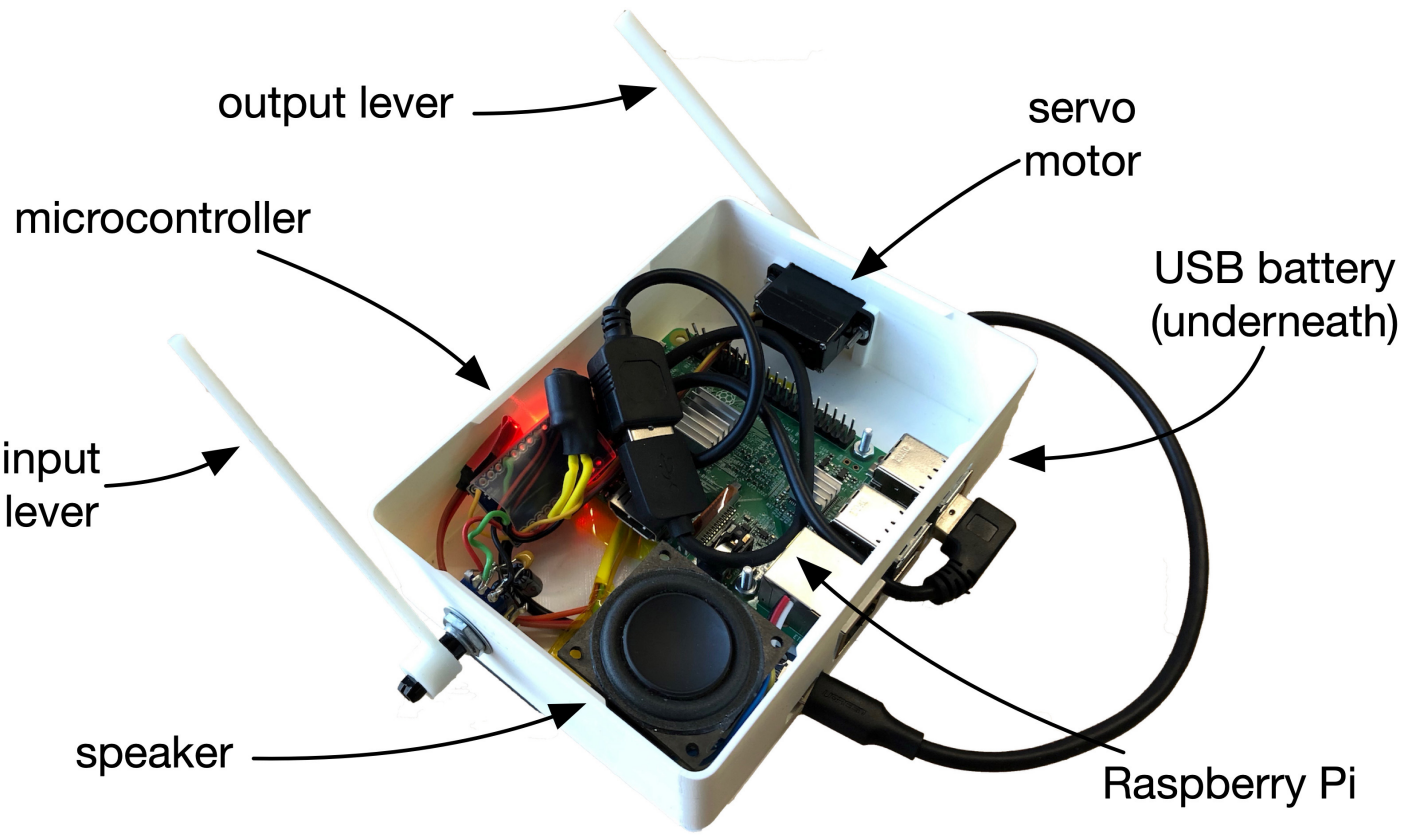
Hardware layout of our self-contained interface. A Raspberry Pi computer provides sound synthesis, model predictions and manages the interactive configuration. Physical input and output is provided by a potentiometer and servo interfaced via a microcontroller. A speaker for audio and USB battery are also included.

The physical design of EMPI is focused on hand-held and self-contained interaction. The 3D-printed enclosure includes a Raspberry Pi model 3B+, one lever for input, a speaker and servo-controlled lever for physical output. A 5,000 mAh USB power bank is attached to the base of the enclosure. The input and output levers are interfaced to the Raspberry Pi through its USB ports and a small ATmega 32U4 microcontroller board. The speaker and a small amplifier is connected directly to the Raspberry Pi's audio output. A system diagram shows these components in Figure 3.

The software aspects of the system provide musical interaction and prediction capabilities. The most important of these is a low-level internal model of performer interactions: a sequence of real-valued potentiometer positions, along with a time-delta value. To model this data, we use a 2D mixture density RNN that predicts the position, and the time, of the next user input. Various trained models can be used with this network based on either real-world or synthetic training data. It should be noted that RNN predictions are computed by the EMPI's Raspberry Pi, not an external system.

The prediction model is implemented in Python using TensorFlow, and applies a special case of our Interactive Music Prediction System (IMPS) which has been previously described (Martin and Torresen, 2019). The IMPS system contains the predictive MDRNN model, and communicates with Pure Data over OSC to receive user interactions and send sound and servo commands. Pure Data synthesizes the sound output and communicates with the microcontroller using MIDI over USB. This system is configured for call-and-response performance. When the performer is playing, their interactions are used to condition the MDRNN's memory state. If they stop playing (after a threshold of 3 s), the MDRNN attempts to continue where they left off, generating more interactions until the performer plays again. The EMPI's hardware design and software, including trained models, are open source and can be found online (Martin, 2019a).

### 3.1. Predictive Model

The EMPI uses a mixture density recurrent neural network to predict future input on the lever. This architecture combines a recurrent neural network with a mixture density network (MDN) (Bishop, 1994) that transforms the output of a neural network to the parameters of a mixture-of-Gaussians distribution. Real-valued samples can be drawn from this distribution, and the number of mixture components can be chosen to represent complex phenomena. The probability density function (PDF) of this distribution is used as an error function to optimize the neural network. In contrast, the softmax layer used in many music RNNs parameterizes a categorical distribution between a set number of discrete classes.

The expressive capacity of MDRNNs has been previously exploited to generate creative data, such as handwriting (Graves, 2013) and sketches (Ha and Eck, 2017). This architecture has only recently been applied to musical interaction data, for instance in *RoboJam* to continue musical touchscreen interactions (Martin and Torresen, 2018), and in *IMPS* as a general model for musical interaction data (Martin and Torresen, 2019). For the EMPI interface, an MDRNN model has the advantage of delivering real-valued samples for lever position and time, as well as a tuneable learning capacity in terms of the RNN configuration (width and number of LSTM layers) and the number of mixture components. This allows us to generate movements in absolute time and to potentially learn complex behaviors from the lever movements.

**Figure 4:**
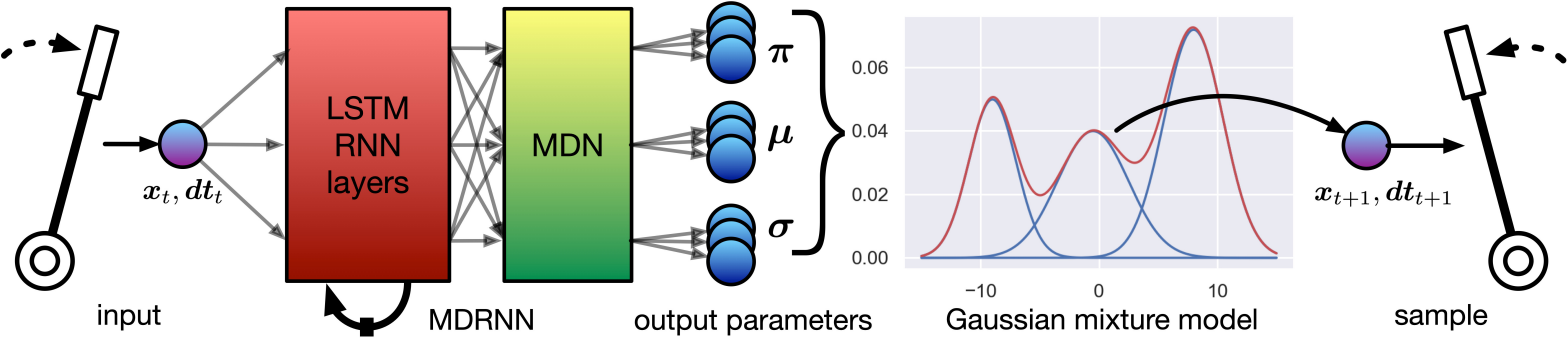
The EMPI's internal model uses a 2D mixture density recurrent neural network (MDRNN) with one dimension predicting the input value and the second predicting time deltas between each movement.

EMPI's MDRNN is a special case of the one described in *IMPS* (Martin and Torresen, 2019), and is illustrated in Figure 4. The neural network has two inputs. One input is for the current lever position (*x*_*t*_), and the other for the time since the previous movement (*dt*_*t*_). These inputs are fed through two layers of long short-term memory (LSTM) units and into the MDN layer which outputs the mixture parameters. Each of the *K* components of the mixture is a bivariate Gaussian distribution with a diagonal covariate matrix with centers (μ_*xk*_, μ_*tk*_) and scales (σ_*xk*_, σ_*tk*_). A set of mixing parameters (π_1_, …, π_*K*_), forms a categorical distribution between the mixture components. In our case, we set the number of mixture components *K* = 5 following previous work (Martin and Torresen, 2019).

**Figure 5:**
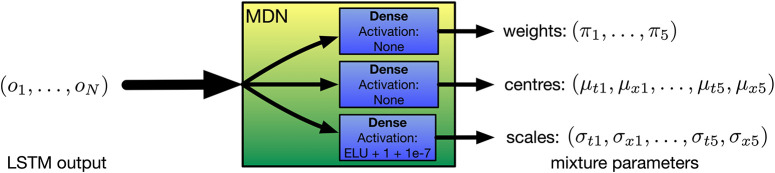
Detail of the EMPI's MDN layer. Three parallel dense layers transform the output of the LSTM units into the parameters of a mixture of bivariate Gaussian probability distributions.

The MDN layer is provided by the Keras MDN Layer (v0.2.1) library (Martin, 2019b). This layer transforms the outputs of the LSTM layers into appropriate parameters to form the mixture distribution (see Figure 5). The outputs of the LSTM layers are fed into parallel dense layers that output the centers, scales, and weights of the mixture distribution, respectively. No activation function is used for the centers and weights. The exponential linear unit (ELU) activation function (Clevert et al., 2016) is used for the scales, with the output offset by 1 + 10^−7^. This ensures that the scales are positive and non-zero while providing gradients at very small values (as recommended by Brando, 2017). To train this neural network, the PDF of the mixture model is constructed using Mixture and MultivariateNormalDiag distributions from the TensorFlow Probability library (Dillon et al., 2017) to provide a likelihood function that the training target was drawn from the mixture distribution predicted by the neural network. The negative log of this likelihood can be used as a loss value for gradient descent to optimize the neural network's weights. Further discussion of this procedure can be found in Bishop's work (Bishop, 1994).

To sample from the parameters output by the MDRNN, first, a mixture component is chosen by sampling from the categorical distribution. Then, this chosen mixture component is sampled to produce an output value. Similarly to other generative RNNs, the sampling diversity, or temperature, can be altered to draw more or less conservative choices. The π_*k*_ form a categorical model that can be adjusted with the usual temperature modification in the softmax function (Hinton et al., 2015, see Equation 1). The covariance matrix can also be scaled to produce a similar effect. This process yields a sample (*x*_*t*+1_, *dt*_*t*+1_), representing a prediction of the next lever position and time at which it could occur. By feeding this sample back into the MDRNN, a continuous stream of lever movements can be generated.

### 3.2. Sound Design

The digital synthesis routine for EMPI runs in Pure Data so a variety of mappings between lever motion and output sound are possible. In our configuration, Pure Data receives one value from the input lever (its position as a MIDI continuous control value), and one from the predictive model's virtual lever. This data is only sent when either lever's position changes, this is similar to the implementation of a fader on a MIDI control surface. We chose to use the lever positions to control pitch. The amplitude of the sound is controlled by an envelope that is only sustained as long as the lever continues to move. This means that rhythmic performance is possible (albeit with small glissandi) by tapping the lever slightly while allowing the sound to diminish in between each movement.

We experimented with controlling a variety of sounds from the levers, such as simple tones, plucked strings (reminiscent of a harp glissando), sample playback, and formant synthesis. For this research, we settled on a simple 4-operator FM synthesis routine with a slight change to the tone controlled by having separate envelopes on modulation and carrier oscillators. Similarly, while it is possible to have dramatically different sounds on the input and output levers, we used the same synth routine (separate voices), with the EMPI's virtual lever tuned one octave lower. This arrangement allows the sounds to be distinguished as different voices, but recognized as coming from the same source.

### 3.3. Data

**Figure 6:**
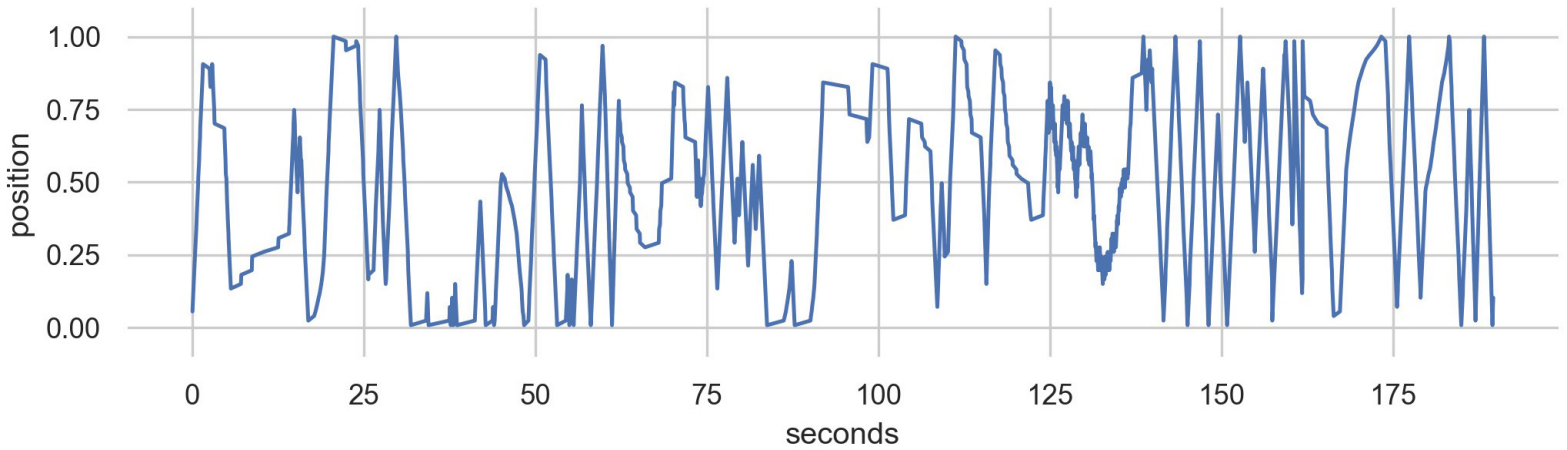
Excerpt from a 10-min human-sourced improvisation with the input lever. This performance was part of the training data for the EMPI's MDRNN model.

We have experimented with models based on three sources of training data: (1) a collection of solo improvised recordings using the EMPI; (2) synthetic data generated from simple waveforms; and (3) uniform noise. The human-sourced data was collected on the EMPI hardware in “human only” mode where the human input was directly linked to a synthesized sound with no input from the internal model. The improvised performances were completely unconstrained and included data from the entire input range of the lever, periods of no interaction (rests), as well as sweeps and movements in different speeds and rhythms. The improvisation was performed by the first author and an excerpt example from the data is shown in Figure 6. This training dataset is available as part of the EMPI source code (Martin, 2019a).

**Figure 7:**
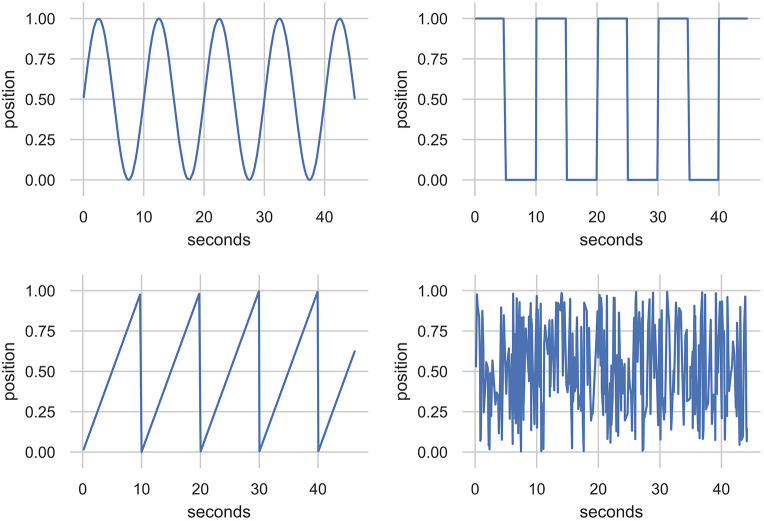
Excerpts from a synthesized data corpus created using stochastically sampled time steps. The function generators are sine, square, and triangle at 0.1 Hz and uniform noise. These data were used as an alternative training data source for the EMPI's MDRNN model.

The synthetic data was generated to represent plausible lever motions in repetitive patterns. To generate these, a sequence of time-steps was drawn stochastically from a normal distribution with mean and standard deviation identical to the human-sourced improvisation^1^. This sequence of time-steps was then fed through sine, square, and triangle wave functions with frequencies at five steps between 0.1 and 1.1 Hz to generate the input values. In total, 10,000 datapoints were generated for each function and frequency resulting in 150,000 total datapoints. The noise data associated a uniformly sampled random number (between 0 and 1) for each of 30,000 time-steps drawn by the same method. Excerpts from the data generated by sine, square, and triangle waves, as well as noise, are shown in Figure 7.

The three sources of data were used to train separate models for the EMPI that are used in the experiments described in section 4. The rationale for using three different models was to explore the creative utility of models based on both human-sourced and synthetically generated data. While the synthetic data is a simple behavior it could potentially represent an appealing and recognizable movement to a performer. In contrast, the noise dataset was not intended to be appealing, rather it was intended to have no recognizable behavior.

## 4. Evaluation

Our evaluation of EMPI is focused on the generative potential of the ML models embedded in the device, and the experience of human performers who interact with it. We first discuss the ML models in the abstract and then describe the results of a human-centered experiment with the EMPI where twelve participants each perform six improvisations under different experimental conditions.

### 4.1. Machine Learning Models

In this section we evaluate the performance of the mixture density RNN architecture and three models applied in the EMPI system. We performed a small training experiment to ascertain an appropriate size of model for the datasets that we used, and generated unconstrained performances from each model to observe what its behavior might be like in performances.

#### 4.1.1. Training

Previous research has suggested that smaller MDRNNs—i.e., with 64 or even 32 LSTM units in each layer, might be most appropriate for modeling small amounts of musical data for integration into an interactive music system (Martin and Torresen, 2019). We trained EMPI's MDRNN models with 32, 64, 128, and 256 units in each LSTM layer to ascertain the best accuracy in terms of reproducing held-out examples from the dataset. Each candidate model used two layers of LSTM units and was trained on sequences that were 50 datapoints in length. Training was conducted using the Adam optimizer with a batch size of 64 and with 10% of training examples held out for validation. For each model, the number of mixture components was held static at 5.

The human dataset contained 75,262 interaction events, corresponding to 65 min of interaction with the EMPI system. The noise dataset included 30,000 interaction events, and the synth dataset included 150,000 interaction events to allow for 10,000 points with each of the 15 signal variations.

**Figure 8:**
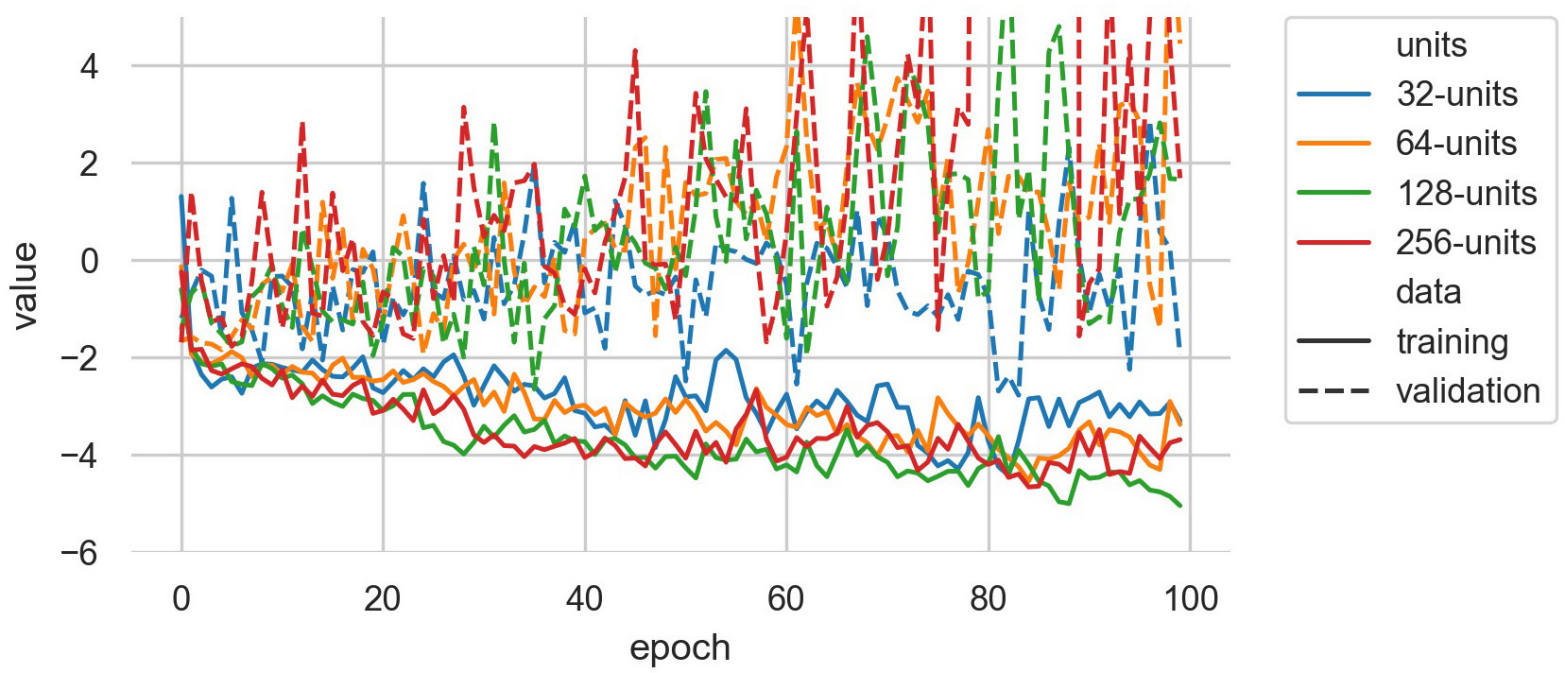
Training data loss and validation data loss while training the human-sourced EMPI model with different size MDRNN architectures. The 32-LSTM-unit MDRNN produced the lowest validation loss and this architecture was used for all EMPI models.

The training and validation set loss over this training process for the human dataset are shown in Figure 8. Over the 100 epochs of training on human-sourced data, the 32-unit MDRNN produced the lowest validation loss. For this reason, and also out of concern for speed of computation on the Raspberry Pi, this size of MDRNN was chosen for our experiments below. The noise and synth models used the same size MDRNN. To avoid overfitting, for each dataset we selected the model with the lowest validation loss achieved during these 100 epochs of training. These models were used for the generation experiments below and in our performer study.

#### 4.1.2. Generation

**Figure 9:**
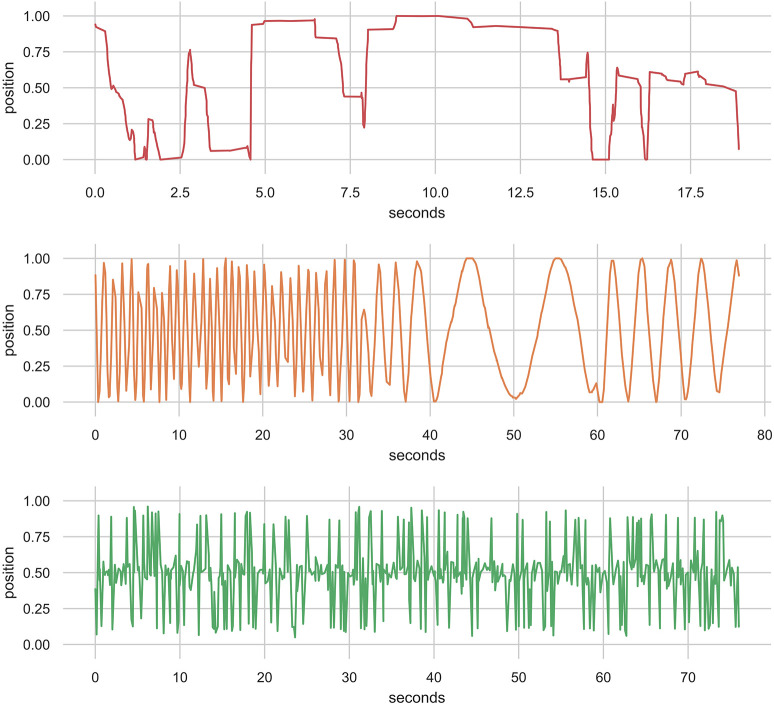
500 Datapoints from the 32-unit MDRNN models in generation mode starting with an uninitialized memory state and a random starting point. The human-, synthetic-, and noise-based models are shown from top to bottom.

To demonstrate the potential output of the RNN models we generated sample performances in an unconstrained manner—starting with an uninitialized memory state and random first value, and linking output to input for 500 prediction steps. Temperature settings of 1.1 for the categorical distribution and 0.1 for the multivariate Gaussian's covariate matrix were chosen by trial-and-error. The results of this experiment are shown for each of the three models (human, synthetic, and noise) in Figure 9.

The output of the human model seems comparable with the human-sourced dataset (see Figure 6). The MDRNN captures a mix of behaviors, such as full back-and-forth motions, small fast movements, and stepping motions with pauses in between movements. The synth model produced output that, similarly to the training data, moves back-and-forth through the whole range of motion with the ability to change its rate of movement. The wave shape seems to change somewhat, but does not deviate from a roughly sinusoidal pattern. The noise model produces unpredictable patterns as expected. Rather than generate uniformly random outputs over the range of the motion, it seems to alternate between the upper and lower extremes with random movements around the middle.

**Figure 10:**
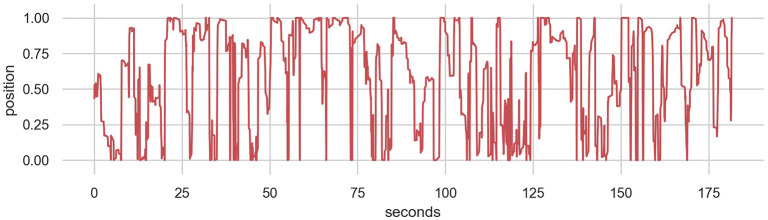
4,500 Datapoints from the 32-unit MDRNN trained on human data resulting in 180 s of performance.

One notable difference between the models is that the human model produces movements at a finer temporal granularity. While 500 samples yields 70 s of movement from the noise and synth models, only 20 s is produced from the human model. This difference becomes apparent in performance with these models as the human model moves much more smoothly than the other two. A longer performance with the human model, produced by sampling 4,500 datapoints, is shown in Figure 10. This shows that the model often focuses on particular areas of the control range for around 10 s before changing to back-and-forth behaviors or moving to a different location. While the long-term structure of the real human performance is not represented, the local structure seems to be reasonably convincing even with this small MDRNN model.

Performance with the three models (see video in Supplementary Material) shows that the noise model produces a consistent but unpredictable pattern, unaffected by any user input. The synth model starts where the user stops, and continues back-and-forth motion. This model can be controlled somewhat by feeding in particularly fast or slow movements, which are matched by the MDRNN model. The human model generates smoother movements that sounds most like normal user inputs. Although it starts in the same location as the user, it seems more difficult to control with different styles of playing than the synth model. All three models appear to be stable and computationally tractable for extended performances on the EMPI's Raspberry Pi.

### 4.2. Performer Study

A study with performers was undertaken to ascertain the effects of the three different models and the absence or presence of physical feedback on their perception of the musical interaction experience. The study took the form of a structured improvisation session where participants performed six short improvisations with the EMPI system under different conditions.

Two independent factors were explored in this study. The first was the *model* that the EMPI device used to make predictions; the three models tested were trained with either human-, synthetic-, or noise-sourced data. The second factor was the *feedback* with the physically-actuated arm either enabled or disabled. These conditions were combined leading to six instrument states and each participant improvised under each of these. The study can be characterized as a two-factor within-groups experiment.

#### 4.2.1. Participants

Participants for the study were recruited from the music and computer science communities at the Australian National University. Twelve respondents (six female, six male) were chosen to participate based on availability and experience with musical performance.

#### 4.2.2. Procedure

**Figure 11:**
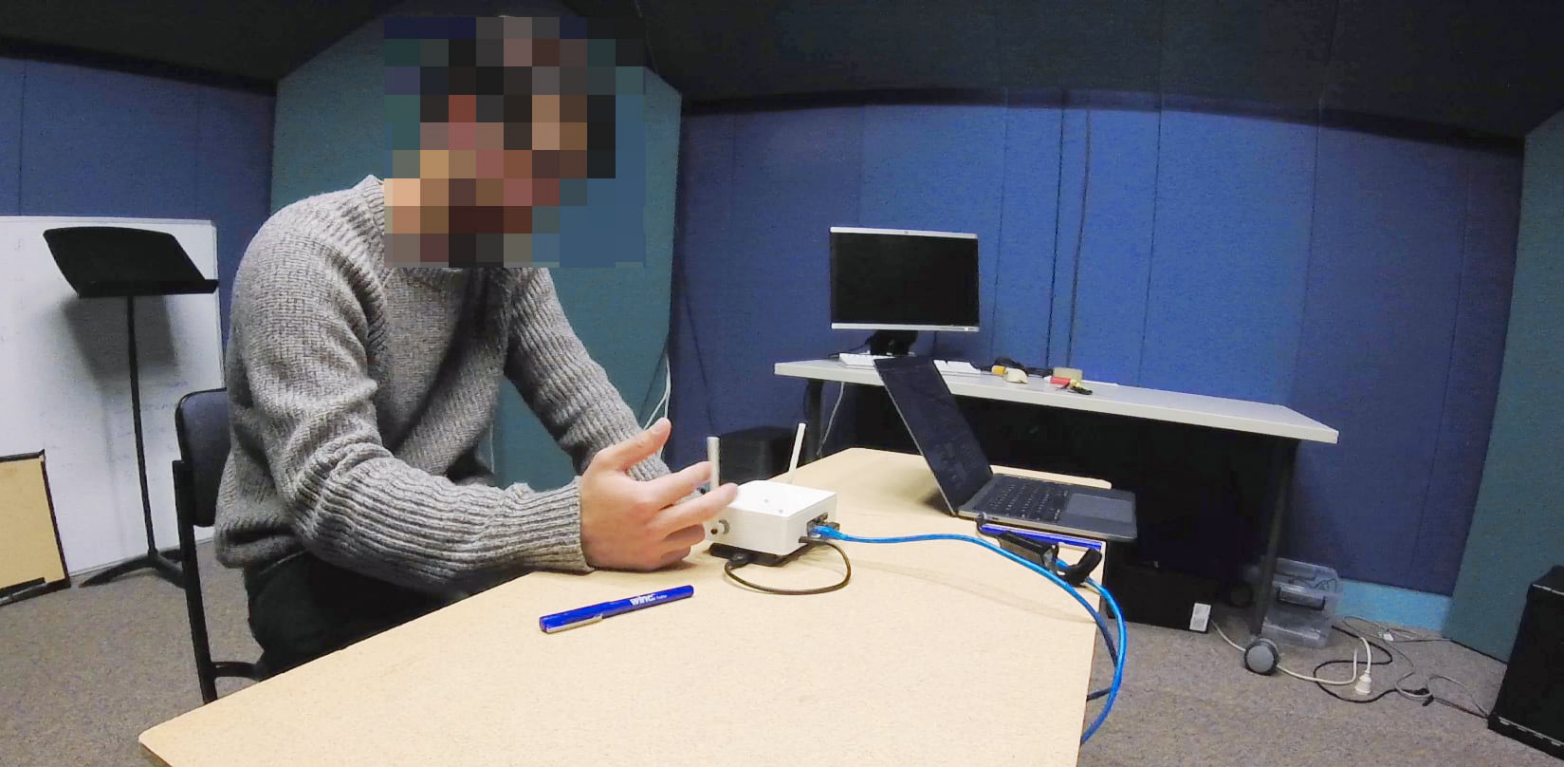
A participant performing with the EMPI during a study session.

The study sessions took the structure of research rehearsals (Martin and Gardner, 2019) in that the participants were asked to perform six short improvisations with each one followed by a written survey and the whole session concluded with an interview. The study environment is shown in Figure 11. The improvisations were finished when the performer determined that they wanted to stop by signaling the researcher, or at a maximum length of 5 min. Each participant's six improvisations was performed with one of the instrument states. The exposure to different states was ordered following a Williams (1949) design to ensure balance with respect to first-order carryover effects. This required six different orderings, each of which was replicated with two different participants.

The collected data consisted of audio, video, and interaction data recordings of the session, a semi-structured interview at the end of the session, and a short written Likert-style survey after each improvisation. The written surveys had 8 questions with each recorded on a 9-point rating scale with labels only on the extremes and midpoint: “Strongly Disagree” (1), “Neutral” (5), “Strongly Agree” (9). The survey questions were as follows:

1. I understood the ML model's responses (*understood*).
2. The responses were related to my performance (*related*).
3. The responses had a high musical quality (*quality*).
4. The responses showed musical creativity (*creativity*).
5. The responses influenced my playing (*inf-play*).
6. My playing influenced the responses (*inf-resp*).
7. The ML model enhanced the performance (*enh-perf*).
8. The ML model enhanced my experience (*enh-exp*).

#### 4.2.3. Survey Results

**Figure 12:**
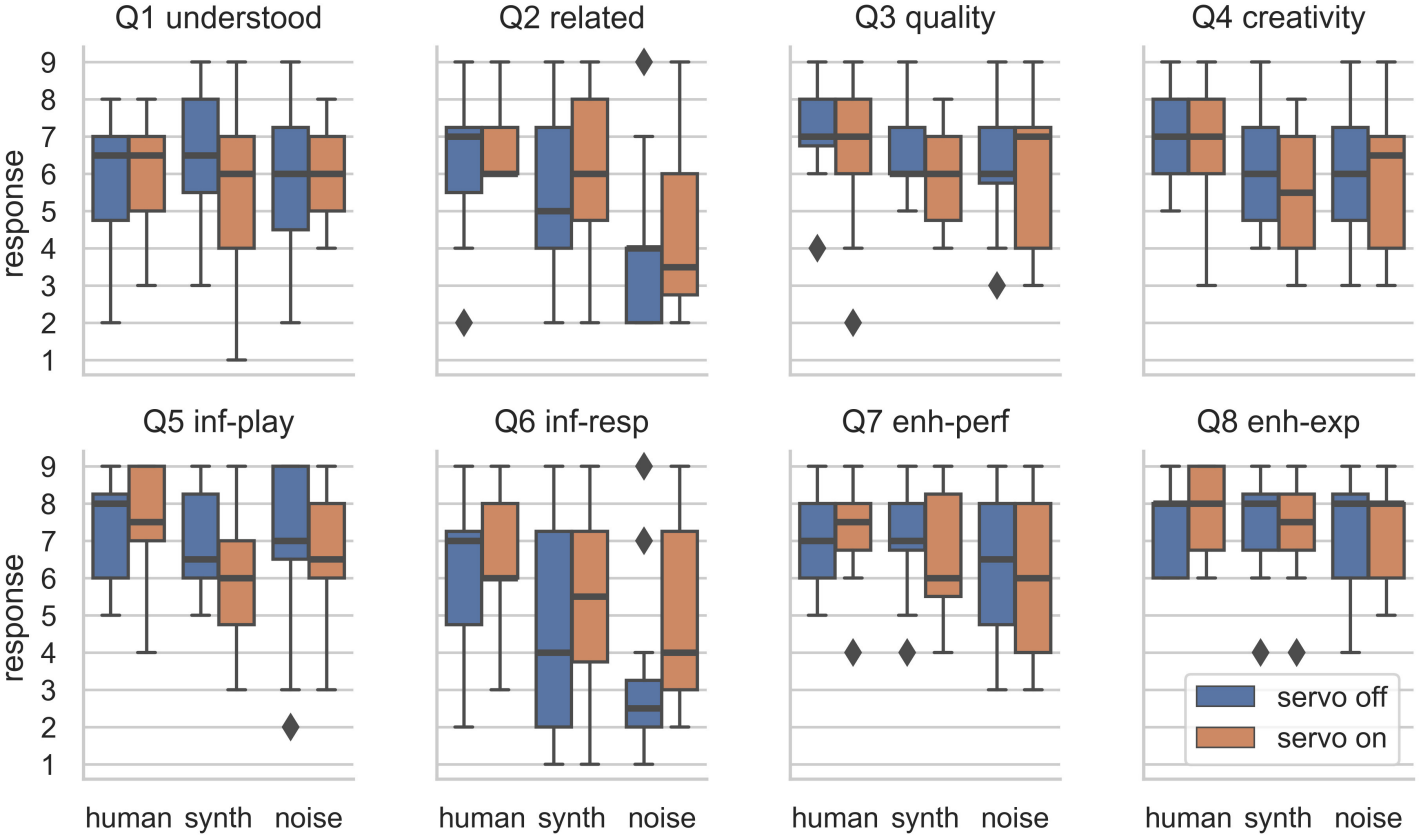
Distribution of responses to the eight survey questions divided by ML model and the presence or absence of the physical lever movement. Outliers are shown as diagonal markers.

The distributions of responses to each question are shown in Figure 12 and the data can be found in the Supplementary Material. Responses to the survey questions were analyzed with an aligned rank transform (ART) and two-way mixed-effects ANOVA procedure. This procedure was used to establish significance of main and interaction effects due to the two factors (*model* and *feedback*). The ART-ANOVA was performed in R using the ARTool library v0.10.6 (Kay and Wobbrock, 2019). This procedure was used as it has been recommended as appropriate for factorial HCI studies with non-parametric data (Wobbrock and Kay, 2016), such as this experiment. *Post-hoc* testing via Holm-corrected paired *t*-tests were performed to establish significant differences between responses to individual conditions.

The ART-ANOVA procedure revealed that the ML model had a significant effect on responses to five of the eight questions; these are shown in Table 1. The model had a significant effect on how participants rated the relation between responses in their performance, the musical creativity of responses, whether responses influenced their playing and vice-versa, and whether the ML model enhanced the performance.

**Table 1:** Survey questions with significant effects due to the ML model.

**Question**	***F***	**Significance**
2. The responses were related to my performance	12.42	*p* < 0.001
4. The responses showed musical creativity	6.87	*p* < 0.01
5. The responses influenced my playing	6.23	*p* < 0.01
6. My playing influenced the responses	6.51	*p* < 0.01
7. The ML model enhanced the performance	3.66	*p* < 0.05

The presence or absence of the servo-actuated lever did not have any significant effects on the survey results. For Question 6, “My playing influenced the responses,” a minor effect [*F*_(1, 55)_ = 2.93, *p* < 0.1] was observed. The distribution of responses here (see Figure 12) show that participants seemed to perceive that they had more influence over the response when the physical actuation was present.

As we detected significant effects of the ML model using the ART-ANOVA procedure, *post-hoc* Holm-corrected paired *t*-tests were used between the results for each ML model to reveal which had led to significantly different responses to these questions. For Question 2, participants reported that the responses were more related to their performance with the human model than the synth model and that the noise model was least related. The differences were significant (*p* < 0.05) for all three models for this question with the human model rated as most related, then synth, then noise. The musical creativity (Q4) of responses was rated significantly higher with the human model than for the other two (*p* < 0.05). The participants reported significantly more influence (Q5) from the human model than from the synth model (*p* < 0.01), but the noise model's influence was not rated significantly differently to the other two. The performers rated their own degree of influence over the human model (Q6) significantly more highly than both the synth and noise models. The noise model was also rated as providing significantly less enhancement (Q7) to the performances than with the human model (*p* < 0.05).

The survey results tell us that performers perceived the ML model as making significant impacts on their performances while the physical feedback only had a minor effect on the participants perception of influence over the responses. The *post-hoc* tests showed that the human ML model's performances were rated as significantly more related to the performers' actions, significantly more creative, and significantly more able to be influenced than the other models. It also influenced the performers' playing significantly more than the synth (but not noise) model. This suggests that the human model had learned enough human-like behavior to interact with the human performers in a natural way. The synth model was rated as performing significantly less related actions than the human model, but was significantly better than the noise model. While the noise model was rated as providing significantly less enhancement to the performances, it did draw some positive ratings, and in particular, was not significantly more or less influential over the player's performance than the other two models.

#### 4.2.4. Interview Results

The interviews following each session were structured around the performers favorite/least favorite condition, whether they preferred the servo on or off, which model they preferred, how they found the interface, and whether they had suggestions for improvement.

Almost all of the participants identified one of the human or synth conditions as their favorite, with physical actuation either on or off. They often reported that these conditions had felt most responsive to their different inputs. Two participants seemed to favor the noise model due to its interesting rhythmic pattern and the fact that it was consistent. Six of the participants indicated that one of the noise conditions had been their least favorite; their main complaint was that they couldn't figure out what the noise model was doing. The other participants chose a human or synth condition as their least favorite. One mentioned disliking the smooth movement of the human model and others disliked the repetitive gestures of the synth model.

Six of the twelve participants preferred to have physical actuation, three preferred not to have actuation, and three had no preference. Some participants preferred to have the visual reinforcement of the model's responses, one noted that it was fun to have it moving, and another that it was similar to eye contact in an ensemble. The servo-detractors felt that it drew their attention away from the sound. One participant even closed their eyes when the servo was turned on.

In general, the participants were able to identify the three distinct models in performance without having been told explicitly during the session. They commented on the idea of exploring the influence they had over the responses as well as taking influence from it. Several participants attempted to lead the models and commented that the synth model seemed to respond most clearly to different kinds of inputs. Some participants were frustrated that the models were most influenced by their training data, rather than the current performance. One suggested implementing something more like a looper. While several participants noticed that the noise model did not respond to their performances, some enjoyed the distinct sound of its performance. Several noted that the human model was distinguished by its “slidy” sound, and one participant thought this made it more expressive than the other models.

In general, participants seemed to enjoy using the EMPI, and several noted that it was “cute” and fun to interact with. Most of the participants commented that they could only “glide” between notes with the lever, rather than skip pitches. In general, this was seen as a limitation when compared with the ability of the ML model to skip between notes. One participant, however, mentioned that they felt they had improved over the session. The participants also saw the focus on pitch control as a limitation and one envisaged controlling other parameters by moving the input lever in other directions. Others suggested extra sounds or controls to fine-tune their input. Although the EMPI was generally seen as unique, one participant compared the EMPI to a flex-a-tone (a novelty percussion instrument) and another to a hurdy gurdy (a string instrument with a crank-driven friction wheel). Several participants saw the strict call-and-response interaction as a limitation, and wanted responses that could overlap with their performance. One suggested reducing the gap between their input and the response to allow for continuous sound.

### 4.3. Discussion

The results of our study reveal variations in how performers perceive the EMPI's machine learning models and interface. The ML model used in each performance had a significant effect on responses to five of the eight survey questions covering the relationship between performance and response, the musical creativity of responses, the amount of influence between the participants' performance and the responses, and the extent to which responses enhanced performances. The human model seemed to produce responses that were most related to the participants' performance and were most creative. This model seemed to influence the performers and receive their influence most readily. On the other hand, several participants reported that the synth model was their favorite in interviews. One participant even favored the noise model.

A complication of this comparison is that the synth and noise models sounded distinct from the participants' performances, primarily due to their quite different temporal behavior. In contrast, the human model sounded more similar to what the performers played. As a result, the human model may have been less memorable at the end of the session. In terms of interaction with the ML models, some participants were concerned with exploring responses, discovering ways to exert control over what the model would do. Others reported drawing inspiration from the ML model's performances, particularly those based on the noise and synth models.

Several participants expressed a desire for the responses to be more directly related to their own performances, perhaps more like a looper, or reflexive instrument (Pachet et al., 2013). In contrast, our MDRNN model (similarly to other RNN-based music systems) has only limited capacity to reflect the performer's input material, and the relationship to the training dataset is much more clear. These participants may have been more interested in ML-systems with on-line training capability. Our study seems to have shown that the performers distinguish between the three models, and see advantages of each one, so a compromise may be to give them control over which ML model is active, emphasizing the strong role of the training data in what they will hear.

The presence or absence of the servo-actuated lever did not have a significant effect on any of the survey questions. The interviews revealed that although half of the participants liked having the servo turned on, the others preferred it off, or had no preference. This split opinion could explain the negative result in the surveys for this effect. It could be that for performers in control of a solo instrument, the physical embodiment of gestures are less important than for an audience watching a performance.

**Figure 13:**
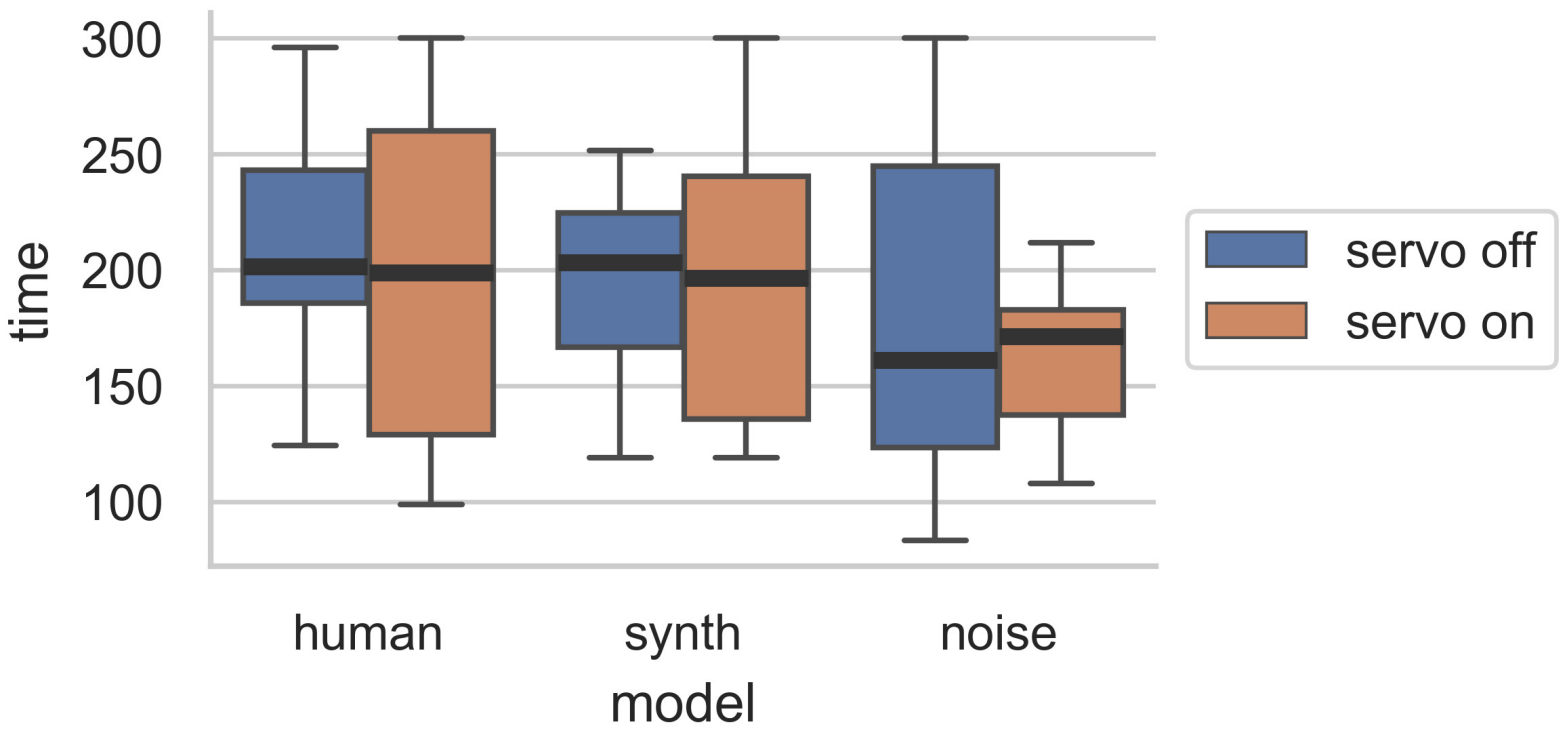
Distribution of performance lengths by experiment condition. The lack of physical actuation resulted in greater variation in the length of performances for the human and synth ML models.

One objective measure of these performances, the length (shown in Figure 13), does show some interesting results related to the servo. For both the human and synth performance, the interquartile range of the length is wider with the servo on than off. For noise, the interquartile range is wider without the servo. An interpretation of these results is that for the more widely favored models, the presence of the servo encouraged some performers, who played for longer, and discouraged others, who stopped performances sooner. The random and unyielding nature of the noise model's performance may have been more apparent with the servo turned on, resulting in shorter performances. It seems that there may yet be an effect due to physical representation of the ML model's behavior in terms of how quickly performers recognize and understand boring responses. A further study could isolate this effect while controlling for differing opinions on physical actuation.

The participants were broadly positive about the EMPI's interface design and interacting with the ML models. They agreed in almost all performances that the ML models had enhanced their experiences, and that the responses showed musical quality and creativity. Although some were frustrated by constraints of the single lever interface, they often overcame these to some extent during performance while attempting to match the behaviors of the ML models. Although the performers generally tried to influence the model's responses, they may have been more influenced themselves. This suggests that the choice of model in EMPI may be more important in terms of suggesting different ways to play the instrument than in picking up the performer's pre-existing musical gestures. Future experiments with EMPI could apply other RNN model architectures or datasets to examine the musical styles they might afford performers.

## 5. Conclusions

In this work, we have examined musical AI through a novel, machine-learning-enabled musical instrument, the embodied musical prediction interface (EMPI). The EMPI system is consciously constrained. This design choice focuses attention toward embodied predictive interaction, where a performer creates music in a call-and-response improvisation with an ML model that can predict physical musical gestures. We use this interface to investigate how different recurrent neural network models are understood and exploited by performers. We also ask whether the physical representation of predictions helps or hinders the performer. While we have examined the generative potential of our ML models, our focus has been on how this system might be used in genuine musical performance. To this end, we conducted a formal, controlled experiment where 12 participants created 72 improvised pieces of music.

Through this study, we found evidence that the ML model's training dataset affects how performers perceive the model's responses, the extent to which they are able to influence it and use it as a source of inspiration. We found that the different performers appreciated different models and that their interest was often drawn to models that were distinct from their playing. Although the survey results often favored the human model, some performers expressed preferences for the model trained on synthetic data and even the model trained on noise. We found that the performers were split on their preference for the physically actuated lever although analysis of the length of the improvised performances suggests that it affects how long the EMPI performance might hold their interest.

These findings suggest that the presence of different ML models can change how users perform with a musical interface. The use of an MDRNN to predict embodied gestural data, rather than musical notes, seems to have added a new dimension of flexibility to our instrument in terms of creating models from synthetic data. The human model sounded most related to the performer's playing, but the two models based on computer-generated data also led to satisfying improvisations. It is entirely feasible to add more custom-designed models to EMPI and to allow musicians to choose which they would like to use, even during the same performance. Our study results suggest that this could lead to new kinds of performances both from the ML response, and the performers' interactions.

While the use of physical actuation was not universally appreciated, overall, the performers reacted positively to the EMPI instrument. Many participants continued to perform and explore the interface and the ML responses up to the 5-min limit of the experimental improvisations. This finding suggests that constrained and gesture-focussed musical instruments can benefit from generative ML interactions that, so far, have often been limited to keyboard-style interfaces. Constrained and self-contained electronic instruments could be an effective way to deploy musical AI systems into broader use by musicians. Physically actuated indicators may be controversial but have the potential to encourage users to explore new ways of operating an interactive music system.

Our work has demonstrated that although simple, EMPI supports a range of musical interactions afforded by the presence of multiple ML models. We also found that while physical actuation of embodied predictions can serve as both an aid and a distraction to different performers, interacting with embodied predictions can enhance a performer's musical experience. Overall, this work contributes new understandings of how musicians use generative ML models in performance backed up by experimental evidence. Our embodied predictive instrument is also a contribution as an open hardware and software system. This research has demonstrated that EMPI can produce compelling music experiences within a lab setting. We argue that EMPI, and future embodied predictive instruments, hold substantial potential for enhancing and enabling musical creativity.

## Data Availability Statement

The survey data and performance durations are available in the Supplementary Material and a video showing the six experimental conditions is available online: https://doi.org/10.5281/zenodo.3521178. The interface and machine learning code for this project is open source and available online: https://doi.org/10.5281/zenodo.3451729.

## Ethics Statement

The studies involving human participants were reviewed and approved by The ANU Human Research Ethics Committee, The Australian National University, Telephone: +61 2 6125 3427, Email: Human.Ethics.Officer@anu.edu.au. The participants provided their written informed consent to participate in this study.

## Author Contributions

CM designed the EMPI interface and machine learning system and conducted the experiments in this work. TN and CM collaborated on the hardware design of the EMPI interface. KG encouraged CM to investigate the system from a self-aware cybernetic system perspective. JT supervised the project and contributed to the research design. All authors provided the critical feedback and helped to shape the research and manuscript.

### Conflict of Interest

The authors declare that the research was conducted in the absence of any commercial or financial relationships that could be construed as a potential conflict of interest.

## Footnotes

^1^The human data above was found to have a mean time-delta of 0.045 s with S.D. 0.184.

## Supplementary Material

Video S1 Overview video of EMPI: the embodied musical predictive interface.

Data Sheet 1 Survey results and durations of performances.
